# Prescription of potentially inappropriate medications among older people with intellectual disability: a register study

**DOI:** 10.1186/s40360-017-0174-1

**Published:** 2017-10-25

**Authors:** Anna Axmon, Magnus Sandberg, Gerd Ahlström, Patrik Midlöv

**Affiliations:** 1 Occupational and Environmental Medicine, Department of Laboratory Medicine, Lund University, 221 00 Lund, SE Sweden; 2 Department of Health Sciences, Lund University, 221 00 Lund, SE Sweden; 3 Center for Primary Health Care Research, Department of Clinical Sciences in Malmö, Lund University, 221 00 Lund, SE Sweden

**Keywords:** Aged, Anticholinergics, Antipsychotics, Benzodiazepines, Middle-aged, Propiomazine, Tramadol

## Abstract

**Background:**

Older people have a greater disease burden and are more likely than younger to be prescribed medications. They are also more sensitive to adverse effects. With this in mind, a range of medications have been suggested inappropriate in this population. People with intellectual disability (ID) have a higher disease burden than the general population, putting them at even greater risk of prescription of such medications. The aim of this study was to describe prescription of potentially inappropriate medications among older people with ID in relation to prescriptions among their age peers in the general population.

**Methods:**

We established an administrative cohort of people with ID (ID cohort; *n* = 7936), using a Swedish national register. A referent cohort from the general population (gPop) was matched one-to-one by sex and year of birth. Data regarding prescription of potentially inappropriate medications were collected from the Swedish prescribed drug register for the years 2006–2012.

**Results:**

People with ID were more likely than the general population to be prescribed medications with anticholinergic effects, intermediate- or long-acting benzodiazepines, and antipsychotics at least once during the study period, and also had more number of years with prescription. Except for benzodiazepines, those in the ID cohort with at least one prescription had larger amounts prescribed than those in the gPop cohort. People in the ID cohort were less likely than the general population to be prescribed non-steroidal anti-inflammatory drugs (NSAIDs). Among those with at least one prescription of NSAIDs, those in the ID cohort had prescriptions during fewer years and in lower amounts than those in the gPop cohort.

**Conclusions:**

Although prescription of potentially inappropriate medications overall is more common among people with ID than in the general population, the opposite pattern is found for medications for pain management. This may be a result of pain being under-recognized and under-treated in this population. Thus, there is a need for training as well as increased knowledge and awareness among care and health care professionals regarding signs of adverse effects and the need of continuous evaluation of treatment in this vulnerable group.

## Background

Aging is associated with ill health [1], reflected in e.g. a greater disease burden [2], and increasing prevalence of frailty [3, 4], chronic pain [5], and concomitant morbidities [6]. As a consequence, older people in the general population consume a disproportionally large part of prescription medications [7]. This is problematic, as older and frail people have been found to be more sensitive to adverse effects [8]. As a result, adverse effects are more common in older than in younger people [9, 10].

Although medical disorders should not be left untreated, some medications should be avoided or used restrictively in elderly populations. In 1991, Beers et al. [11] published criteria for determining potentially inappropriate medication (PIM) use in nursing-home residents. These criteria have since then been extended to all older adults and are continuously revised by the American Geriatrics Society, with the latest revision published in 2015 [12]. However, although several countries – including Sweden [13] – have published guidelines for drug prescription, compliance is low when it comes to older people in the general population [14]. It has been proposed that more than half of older people has at least one prescription of PIMs [15], and some studies suggest that the risk of being prescribed PIMs are greater among women than among men [15, 16].

People with intellectual disability (ID) have a higher disease burden than the general population [17]. Hence, drug prescription is expected to be higher among people with ID than in the general population. However, although some data suggest that frequency of prescribing corresponds to the frequency of chronic conditions [18], others indicate high levels of excessive polypharmacy [18] and off-label use, mainly for antipsychotics [19–21]. With respect to adverse effects, older people with ID may be more sensitive than the general population [22]. Thus, it is important to describe prescription of PIMs among people with ID, in order to provide basis for actions to decrease adverse effects in this vulnerable population.

## Methods

The aim of the present study was to investigate prescription of PIMs in a group of older people with ID in comparison with the same age group in the general population. Moreover, to describe potential differences based on sex, and to assess effects of sex, age, and living in special housing among people with ID. The study is register-based, using three Swedish national registers. The registers were used both to identify the study population and to collect information on exposure and outcome variables.

### Setting

In Sweden, each person planning to stay in the country for at least one year is provided with his or her own unique personal identification number (PIN) at birth or immigration. In all registers described below, records are linked to a specific person using this PIN. Thus, record linking can be done to comprise a database with relevant register data.

The Total Population Register is maintained by the governmental administrative agency Statistics Sweden, with the aim to produce statistics about the Swedish population. The register contains information on life events including birth, marital status, and migration. Updates are transmitted daily from the Swedish Tax Agency.

Swedish people with ID or autism spectrum disorder (ASD) can apply to their municipalities for different measures of support, such as a personal assistant or day-time activities, to be able to live a full-filling life on the same terms as people in the rest of the population. These measures are regulated in the Swedish Act Concerning Support and Service for People with Certain Functional Impairments (aka the LSS) [23]. There are ten forms of assistance specified in the act. One form is supported living in a group home or a service home, henceforth referred to as “special housing”. In both types of special housing, each resident has his or her own apartment and access to common areas. Group homes are staffed around-the-clock, whereas in service homes, service staff is available at all hours, but not always on site. Special housing is available for adults as well as for children and young people. The other forms of support are advice and other personal support, personal assistance, companion service, contact person, relief service, short-term stays away from home, short-term care for schoolchildren over 12, and daily activities. All support and service provided according to the LSS act is documented in the LSS register, which is maintained by the Swedish National Board of Health and Welfare, a government agency under the Ministry of Health and Social Affairs. The register contains information on the individual, such as sex and date of birth, and on the services provided, such as type of service and providing municipality. It does not contain information on any diagnoses of ID or ASD.

The Swedish Prescribed Drugs Register was established in July 2005, and contains information on all dispensed prescriptions to the whole of Sweden’s population. Also this register is maintained by the Swedish National Board of Health and Welfare. Each record contains information on the patient, such as age and sex, the drug, such as substance and dispensed amount, and the prescriber, such as profession and type of care facility. Drugs are defined using the Anatomical Therapeutic Chemical (ATC) classification system [24]. As the name indicates, this system classifies drugs according to anatomical, therapeutic, and chemical group. E.g. all drugs classified in the main group “N” act on the nervous system. The system consists of five levels, where the fifth level identifies the chemical substance. The ATC classification system also includes Defined Daily Dose (DDD) for many drugs [24]. The DDD is the average adult dose used for the main indication of the medicine.

### Study cohorts

The cohort of older people with ID (the ID cohort) was established using the LSS register. Through this register, all people aged at least 55 years and alive at December 31st 2012, and with at least one service registered during this year, were identified (*n* = 7936). The referent cohort (the gPop cohort) comprised people from the general population, one-to-one matched by sex and birth year by using the Total Population Register.

### Outcomes

In 2010, the Swedish National Board of Health and Welfare published a report listing medications that may need extra attention among older people [13]. Intermediate- or long-acting benzodiazepines (N05BA01, N05CD02, and N05CD03), medications with anticholinergic effects (anticholinergics; N05BB, G04, and R06AD), tramadol (N02AX), and propiomazine (N05CM) were listed under the heading “avoid unless special reason for prescription”. All these were included as PIMs in the present study. The report further listed medications where a correct and current indication was particularly important. Of these, we included antipsychotics (N05A excluding lithium, N05AN), as it has been found commonly prescribed among people with ID [19, 25, 26], and non-steroidal anti-inflammatory drugs (NSAIDs; M01A), as there are indications that pain may be under-treated among people with ID [27]. Data on all dispensations of these medications during the period 2006–2012 were collected from the Prescribed Drug Register.

In the main analyses, we compared the ID cohort with the gPop cohort with respect to a) having at least one prescription of each medication during each year, b) number of years with prescription, and c) individual average DDD per medication during years with prescription. These analyses were performed for the whole cohorts, as well as stratified by sex. In secondary analyses, we investigated the effect of age, gender, and living in special housing on prescriptions of PIMs within the ID cohort.

### Statistics

Analyses of dichotomous outcomes (e.g. having at least one prescription) were performed using generalized linear models (GLM), estimating relative risks (RRs) with 95% confidence intervals (CIs). When yearly observations were included in the model, calendar year was used to indicate repeated measures.

P-p-plots revealed that although the original values of individual average DDD were skewed, ln-transformed values were normally distributed. Thus, analyses were performed using Analysis of Variance (ANOVA) on ln-transformed values. As data regarding number of years with prescriptions were skewed both in their original form and after ln-transformation, comparisons were made using the Mann-Whitney U-test.

A two-tailed *p*-value of 0.05 was considered statistically significant. All statistical analyses were performed in IBM SPSS Statistics version 23.

## Results

Of the 7936 individuals included in each cohort, 3609 (45%) were women and 4327 (55%) were men. The age at the start of the study period, i.e. in 2006, ranged between 49 and 90 years, and at the end of the study period between 55 and 96 years.

The percentage of people with prescription each year, as well as the number and percentage of people who were prescribed different medications at least once is described for the whole study period in Table 1. The main results for the different medications/medication groups are presented below. Unless otherwise stated, the results found when comparing the ID cohort to the gPop cohort were consistent when stratifying on sex.

**Table 1:** Prescription of PIMs among people with ID and from the general population

		Yearly % with prescription	Number of years with prescription [n (%)]							Median (range)					
			1	2	3	4	5	6	7	All		Women		Men	
At least one	gPop	27–28	1822 (35)	1093 (21)	678 (13)	501 (9)	366 (7)	352 (7)	465 (9)	*n* = 5277	2 (1–4)	*n* = 2477	2 (1–4)	*n* = 2800	2 (1–4)
	ID	50–52	997 (17)	565 (10)	359 (6)	310 (5)	268 (5)	371 (6)	2921 (50)	*n* = 5791	7 (2–7)	*n* = 2635	6 (2–7)	*n* = 3156	7 (2–7)
	ID vs gPop										<0.001		<0.001		<0.001
Anticholinergics	gPop	4–6	639 (51)	218 (18)	110 (9)	82 (7)	77 (6)	57 (5)	61 (5)	*n* = 1244	1 (1–3)	*n* = 482	1 (1–2)	*n* = 762	2 (1–3)
	ID	9–12	545 (33)	216 (13)	180 (11)	127 (8)	106 (6)	124 (7)	372 (22)	*n* = 1670	3 (1–6)	*n* = 655	3 (1–6)	*n* = 1015	3 (1–6)
	ID vs gPop										<0.001		<0.001		<0.001
Benzodiazepines	gPop	2–3	200 (47)	58 (14)	26 (6)	18 (4)	25 (6)	33 (8)	63 (15)	*n* = 423	2 (1–5)	*n* = 244	2 (1–5)	*n* = 179	2 (1–5)
	ID	9–11	548 (34)	252 (16)	132 (8)	106 (7)	75 (5)	84 (5)	412 (26)	*n* = 1609	3 (1–7)	*n* = 722	2 (1–6)	*n* = 887	3 (1–7)
	ID vs gPop										<0.001		0.006		<0.001
NSAIDs^a^	gPop	16–19	1961 (45)	969 (22)	582 (13)	361 (8)	228 (5)	164 (4)	107 (2)	*n* = 4372	2 (1–3)	*n* = 2092	2 (1–3)	*n* = 2280	2 (1–3)
	ID	9–10	1422 (54)	519 (20)	259 (10)	180 (7)	87 (3)	81 (3)	80 (3)	*n* = 2628	1 (1–3)	*n* = 1331	1 (1–3)	*n* = 1297	1 (1–2)
	ID vs gPop										<0.001		<0.001		<0.001
Propiomazine	gPop	2–3	369 (54)	104 (15)	58 (9)	28 (4)	38 (6)	36 (5)	46 (7)	*n* = 679	1 (1–3)	*n* = 361	1 (1–3)	*n* = 318	1 (1–3)
	ID	7–9	344 (29)	159 (14)	101 (9)	93 (8)	85 (7)	87 (7)	306 (26)	*n* = 1175	3 (1–7)	*n* = 536	3 (1–7)	*n* = 639	3 (1–7)
	ID vs gPop										<0.001		<0.001		<0.001
Tramadol	gPop	4–5	973 (65)	245 (16)	94 (6)	50 (3)	46 (3)	41 (3)	40 (3)	*n* = 1489	1 (1–2)	*n* = 747	1 (1–2)	*n* = 742	1 (1–2)
	ID	3–3	585 (63)	150 (16)	62 (7)	42 (5)	38 (4)	17 (2)	33 (4)	*n* = 927	1 (1–2)	*n* = 488	1 (1–2)	*n* = 439	1 (1–2)
	ID vs gPop										0.187		0.286		0.476
Antipsychotics (excluding lithium)	gPop	1–1	88 (37)	43 (18)	21 (9)	8 (3)	16 (7)	13 (6)	46 (20)	*n* = 235	2 (1–6)	*n* = 116	2 (1–5)	*n* = 119	2 (1–6)
	ID	34–34	217 (7)	140 (4)	94 (3)	93 (3)	108 (3)	194 (6)	2270 (73)	*n* = 3116	7 (6–7)	*n* = 1337	7 (6–7)	*n* = 1779	7 (6–7)
	ID vs gPop										<0.001		<0.001		<0.001

Number of years with prescription of potentially inappropriate medications (PIMs) among 7936 people with intellectual disability (ID) and a sample from the general population (gPop) one-to-one matched on sex and age

^a^Non-steroidal anti-inflammatory drugs

### Potentially inappropriate medications

**Fig. 1:**
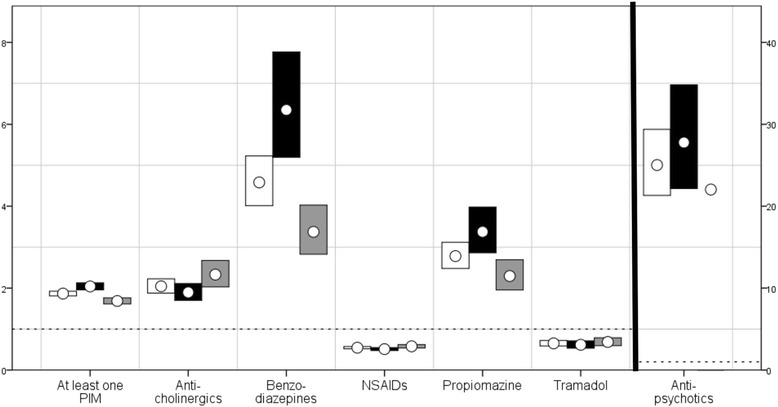
RRs for prescription of PIMs among people with ID compared with the general population. *Risk ratios with 95% confidence interval for prescription of potentially inappropriate medications (PIMs) for people with intellectual disability* vs *a one-to-one age and sex matched sample from the general population*. NSAIDs = Non-steroidal anti-inflammatory drugs; RRs are presented for the whole cohort (white; n=7936), men (black; n=4327), and women (gray; n=3609)

**Fig. 2:**
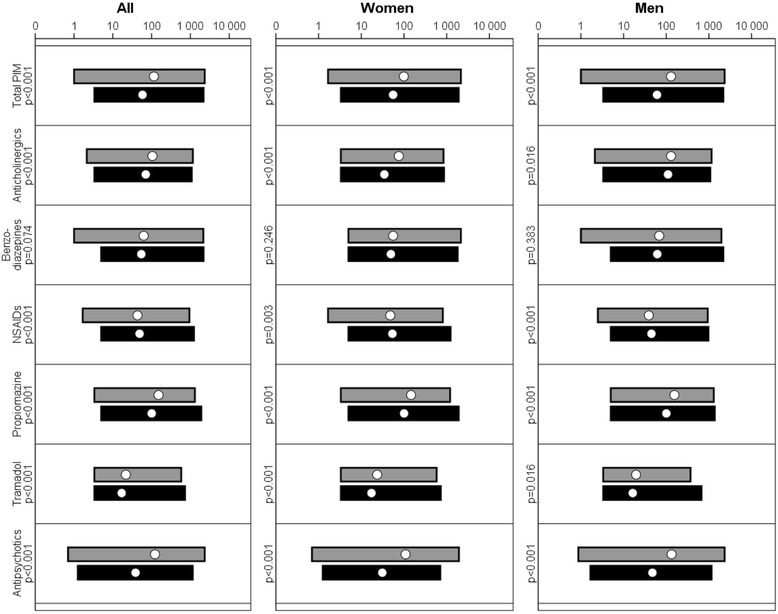
Average DDD of PIMs for people with ID and the general population. *Geometric mean, maximum and minimum of individual average DDD (defined daily dose) for each potentially inappropriate medication (PIM) and cohort, displayed on a logarithmic scale*. NSAIDs = Non-steroidal anti-inflammatory drug; Black = gPop cohort, gray = ID cohort

**Fig. 3:**
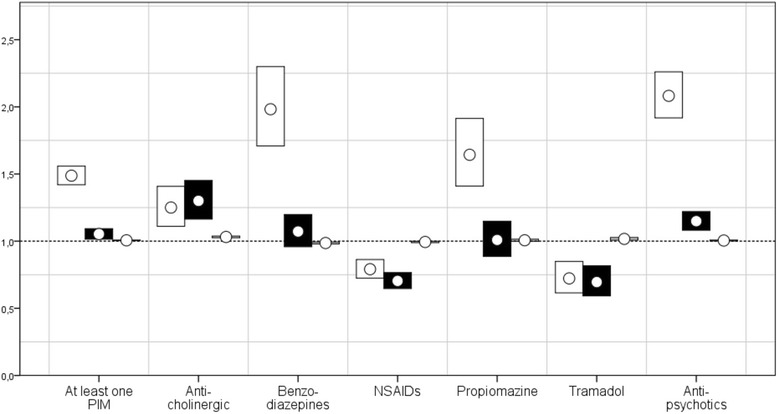
RRs for prescription of PIMs among people with ID. *Risk ratios with 95% confidence interval for prescription of potentially inappropriate medications (PIMs) within a group of people with intellectual disability.* NSAIDs = Non-steroidal anti-inflammatory drugs; White = special housing vs not special housing, black = men vs women, grey = age

There was an almost 90% increased risk for the ID cohort for yearly prescription of at least one PIM (Fig. 1). Half of the people in the ID cohort were prescribed at least one PIM during each year of the study period, compared to 9% in the gPop cohort (Table 1). This resulted in a higher median number of years of prescription in the ID cohort. Among those with at least one prescription, the average DDD was higher in the ID cohort than in the gPop cohort (Fig. 2). Within the ID cohort, male gender, living in special housing, and increasing age was associated with being prescribed at least one PIM (Fig. 3).

### Anticholinergics

People in the ID cohort were more likely to be prescribed anticholinergic medications than those in the gPop cohort (Fig. 1), and the median number of years of prescription was higher in the ID cohort than in the gPop cohort (Table 1). Among those with at least one prescription, the DDD was higher in the ID cohort than in the gPop cohort (Fig. 2). Within the ID cohort, male gender, living in special housing, and increasing age was associated with being prescribed anticholinergics at least once (Fig. 3).

### Benzodiazepines

People in the ID cohort were more likely to be prescribed benzodiazepines than those in the gPop cohort (Fig. 1), and the median number of years of prescription was higher in the ID cohort than in the gPop cohort (Table 1). Among those with at least one prescription, the DDD was similar in the ID cohort and the gPop cohort (Fig. 2). Within the ID cohort, living in special housing, and decreasing age was associated with being prescribed benzodiazepines at least once (Fig. 3). No effect was found for gender.

### NSAIDs

People in the ID cohort were less likely to be prescribed NSAIDs than those in the gPop cohort (Fig. 1), and the median number of years of prescription was lower in the ID cohort than in the gPop cohort (Table 1). Among those with at least one prescription, the DDD was lower in the ID cohort than in the gPop cohort (Fig. 2). Within the ID cohort, female gender, not living in special housing, and decreasing age was associated with being prescribed NSAIDs at least once (Fig. 3).

### Propiomazine

People in the ID cohort were more likely to be prescribed propiomazine than those in the gPop cohort (Fig. 1), and the median number of years of prescription was higher in the ID cohort than in the gPop cohort (Table 1). Among those with prescription of propiomazine, the DDD was higher in the ID cohort than in the gPop cohort (Fig. 2). Within the ID cohort, living in special housing was associated with being prescribed anticholinergics at least once (Fig. 3). No effects were found for gender or age.

### Tramadol

People in the ID cohort were less likely to be prescribed tramadol than those in the gPop cohort (Fig. 1), but there was no difference between the ID and gPop cohorts with regards to number of years with prescription (Table 1). Among those with at least one prescription, the DDD was higher in the ID cohort than in the gPop cohort (Fig. 2). Within the ID cohort, female gender, not living in special housing, and increasing age was associated with being prescribed anticholinergics at least once (Fig. 3).

### Antipsychotics

People in the ID cohort were more likely to be prescribed antipsychotics than those in the gPop cohort (Fig. 1). The results were consistent among men, but the number of women with prescription was too low to draw any conclusions. The median number of years of prescription was higher in the ID cohort than in the gPop cohort (Table 1). Among those with at least one prescription, the DDD was higher in the ID cohort than in the gPop cohort (Fig. 2). Within the ID cohort, male gender, living in special housing, and increasing age was associated with being prescribed antipsychotics at least once (Fig. 3).

## Discussion

People in the ID cohort were more likely than those in the gPop cohort to be prescribed all PIMs, except for those used in the management of pain. The same pattern, i.e. with increased risk of prescription of all PIMs except those used for pain management, was found when comparing those living in special housing to those living elsewhere within the ID cohort. Among people with ID, men were more likely than women to be prescribed anticholinergics and antipsychotics, but less likely to be prescribed medications for pain management. Increased risk of prescription was generally associated with prescription of higher doses and for longer periods of time. The exception was tramadol, where those with fewer prescriptions had higher doses.

Besides the size of the cohort of older people with ID, the present study has a major strength in that data on prescriptions were collected from a nationwide register covering all prescribed medicines dispensed at all pharmacies in Sweden. However, when interpreting the results, one must be aware that the register does not contain information on over-the-counter purchases, nor does it provide data on medications requisitioned for use in hospital wards. Moreover, data on medications administered in day care and outpatient care at hospitals are partially missing. Thus, the use of any such medications would be underestimated. If this underestimation differs between the ID and gPop cohorts, the risk estimates may also be compromised such that a greater underestimation of use in the ID cohort than in the gPop cohort would lead to an underestimation of the risk when comparing the ID cohort to the gPop cohort. The opposite result would follow if underestimation of use is more likely to occur in the gPop cohort than in the ID cohort.

We used inclusion in the LSS-register, i.e. having received support and service according to the LSS act, as a proxy for ID. There are two downsides with this. One is that the LSS register does not contain diagnoses but only registrations of which support and services have been provided. Thus, we have no information on type and severity of ID. Moreover, it is possible to be included in the LSS-register based on a diagnosis of ASD. Thus, the ID cohort may be “diluted” with people without ID but with ASD. In order to assess a possible impact of this, we defined subgroups within the ID cohort by using diagnoses from the Swedish National Patient Register: ASD-diagnosis only (*n* = 189), and ID-diagnosis only or in combination with ASD-diagnosis (*n* = 2147). The remaining 5600 people in the ID cohort had either not had a physician visit recorded during 2002–2012, or had other diagnoses than ASD or ID recorded for all visits. As the number of people with ID, alone or combination with ASD, is far greater than the number with ASD without ID, the impact of including also those with ASD should be minor.

In the present study, people with ID had higher risks of being prescribed PIMs than the general population. There may be several explanations for this. The most intuitive one is that the prescription rate corresponds to disorder prevalence. For example, anticholinergics are used in the treatment of gastrointestinal and respiratory disorders, which are common among people with ID [17, 28, 29]. Moreover, people with ID are more likely to have co-morbidity than the general population [17] and co-morbidity is correlated with use of PIMs [30]. The same is true for mental disorders [31, 32]. However, in the case of benzodiazepines [33] and propiomazine [34], effective alternative treatments with less potential adverse effects are available. Thus, the large increased risk of prescription of these medications among older people with ID is a cause of concern, and more likely to reflect lack of awareness among prescribing health care professionals than a high disease burden among people with ID.

Besides various disorders, there are other factors associated with both ID and use of PIMs, and thereby possible links between these. These include low socioeconomic status [35–37] and unhealthy behavior, such as a sedentary lifestyle and improper nutrition [38, 39]. However, a more significant contributor is most likely the use of PIMs, such as antipsychotics, to treat challenging behavior among people with ID [19–21]. Also, prescription errors (i.e. prescription not in concordance with current standards) are frequent among older people with ID, with too high dosage and unnecessary drug therapy being the most common [40].

The high prescription among people with ID may also be due to long-term use without proper evaluation of, or failure to recognize, adverse effects. For some of the PIMs investigated in the present study, i.e. antipsychotics, benzodiazepines, and anticholinergics, a common adverse effect is cognitive impairment [12, 13, 41], which may be difficult to detect among people with ID. Others have adverse effects that are overlapping with disorders already common among people with ID, such as antipsychotics and constipation [12, 28]. Thus, unless particular attention is paid to potential adverse effects of PIMs in this group of people, they may go undetected, leading to even more deterioration of health.

Prescription of PIMs may be reduced by e.g. interventions targeted towards physicians [42] or using computerized warning systems [43]. These are methods not depending on the ability of the patient, and should thus be possible to use also among people with ID. However, medication reviews have been found feasible to perform among people with ID [44, 45]. Hence, higher prescription rate of PIMs, such as that found in the present study, should be avoidable. This might be achieved e.g. by educating care and health care professionals regarding the particular needs of people with ID, and the importance of continuous monitoring of adverse effects and re-evaluation of treatment regime.

A potential cause of concern is the lower prescription rate of medications used to treat pain, i.e. NSAIDs and tramadol, among people with ID compared with the general population. That we consider this possibly worrisome may be counterintuitive, as we have claimed these to be medications that should be avoided. If the lower prescription rate is an effect of less pain among people with ID, or that they are prescribed other – better – treatment regimes, it would be a positive result. However, this is most likely not the case [46–48]. As people with ID, and especially profound ID, are less able to conceptualize and communicate their symptoms [49], it is more likely that the lower prescription rate is due to failure to recognize the pain rather than there being none [27]. This is supported by other studies, where NSAIDs and opioids were found to be less prescribed among those with cognitive impairment [50, 51].

Within the ID cohort, increasing age was associated with prescription of at least one PIM, which is in agreement with previously published data [52–54]. In the general population, adverse effects associated with PIMs have been found to be more common among older people than in younger populations, and also to occur at lower doses [41, 55]. Notwithstanding the burden placed on the individual, the increased health care consumption due to use of PIMs [56–60] is also associated with increased health care costs for the society [61]. Thus, to be aware of and monitor adverse effects is especially important in older patients with ID.

When comparing people with ID living in special housing to people with ID living elsewhere, the same pattern as when comparing people with ID to the general population emerged. This may not be surprising, as those living in special housing most likely have more health related problems. However, the increased risk of prescription of e.g. benzodiazepine could also be a result of a desire to keep residents more easily manageable.

In the present study, the risk of prescription of PIMs associated with ID was higher among men than among women. In the general population, women are more likely than men to be prescribed PIMs [52, 62–64]. However, in the present study, they were less likely to be prescribed PIMs. Thus, the lower risk associated with ID among women compared to men is probably driven both by a higher prescription among women in the general population and a lower prescription among women among people with ID. Why opposite gender patterns are found in the general population and among people with ID needs to be further investigated.

## Conclusions

PIMs are more common among people with ID than in the general population. They are also prescribed for longer periods and at higher doses. However, medications for pain management are less prescribed to people with ID, possibly since pain is under-recognized and under-treated in this population. There is a need for training and increased knowledge among care professionals regarding symptoms and signs of adverse effects. Also, for an awareness among health care professionals of the need of continuous evaluation of treatment in this vulnerable group.

## Abbreviations

ANOVA: Analysis of Variance
ASD: Autism Spectrum Disorder
ATC: Anatomical Therapeutic Chemical
CI: Confidence Interval
DDD: Defined Daily Dose
GLM: Generalized Linear Model
gPop: General Population
ID: Intellectual Disability
LSS: Act Concerning Support and Service for People with Certain Functional Impairments
NSAIDs: Non-steroidal anti-inflammatory drugs
PIM: Potentially inappropriate medication
PIN: Personal Identification Number
RR: Relative Risk

## References

[1] WHO. World report on ageing and health. 2015.

[2] Veras R (2009). Population aging today: demands, challenges and innovations. Rev Saude Publica.

[3] Rockwood K, Song X, Mitnitski A (2011). Changes in relative fitness and frailty across the adult lifespan: evidence from the Canadian National Population Health Survey. CMAJ.

[4] Gill TM, Gahbauer EA, Allore HG, Han L (2006). Transitions between frailty states among community-living older persons. Arch Intern Med.

[5] Mansfield KE, Sim J, Jordan JL, Jordan KPA (2016). Systematic review and meta-analysis of the prevalence of chronic widespread pain in the general population. Pain.

[6] Corona G, Lee DM, Forti G, O'Connor DB, Maggi M, O'Neill TW, Pendleton N, Bartfai G, Boonen S, Casanueva FF (2010). Age-related changes in general and sexual health in middle-aged and older men: results from the European male ageing study (EMAS). J Sex Med.

[7] Willlams CM (2002). Using medications appropriately in older adults. Am Fam Physician.

[8] Hubbard RE, O'Mahony MS, Woodhouse KW (2013). Medication prescribing in frail older people. Eur J Clin Pharmacol.

[9] Beijer HJ, de Blaey CJ (2002). Hospitalisations caused by adverse drug reactions (ADR): a meta-analysis of observational studies. Pharm World Sci.

[10] Vrdoljak D, Borovac JA (2015). Medication in the elderly - considerations and therapy prescription guidelines. Acta Med Acad.

[11] Beers MH, Ouslander JG, Rollingher I, Reuben DB, Brooks J, Beck JC (1991). Explicit criteria for determining inappropriate medication use in nursing-home residents. Arch Intern Med.

[12] American_Geriatrics_Society (2015). American Geriatrics Society 2015 updated beers criteria for potentially inappropriate medication use in older adults. J Am Geriatr Soc.

[13] Socialstyrelsen. Indicators of good drug therapy in the elderly [In Swedish: Indikatorer för god läkemedelsterapi hos äldre]. 2010.

[14] Etchepare F, Pambrun E, Verdoux H, Tournier M. Trends in patterns of antidepressant use in older general population between 2006 and 2012 following publication of practice guidelines. Int J Geriatric Psychiatry. 2016; 10.1002/gps.4536 27357262

[15] Guaraldo L, Cano FG, Damasceno GS, Rozenfeld S (2011). Inappropriate medication use among the elderly: a systematic review of administrative databases. BMC Geriatr.

[16] Buck MD, Atreja A, Brunker CP, Jain A, Suh TT, Palmer RM, Dorr DA, Harris CM, Wilcox AB (2009). Potentially inappropriate medication prescribing in outpatient practices: prevalence and patient characteristics based on electronic health records. Am J Geriatr Pharmacother.

[17] Sandberg M, Ahlström G, Kristensson J (2017). Patterns of somatic diagnoses in older people with intellectual disability: a Swedish eleven year case-control study of inpatient data. Journal of applied research in intellectual disabilities : JARID.

[18] O'Dwyer M, Peklar J, McCallion P, McCarron M, Henman MC (2016). Factors associated with polypharmacy and excessive polypharmacy in older people with intellectual disability differ from the general population: a cross-sectional observational nationwide study. BMJ Open.

[19] Doan TN, Lennox NG, Taylor-Gomez M, Ware RS (2013). Medication use among Australian adults with intellectual disability in primary healthcare settings: a cross-sectional study. J Intellect Develop Disabil.

[20] Deb S, Unwin G, Deb T (2015). Characteristics and the trajectory of psychotropic medication use in general and antipsychotics in particular among adults with an intellectual disability who exhibit aggressive behaviour. J Intellect Disabil Res.

[21] Tsiouris JA, Kim SY, Brown WT, Pettinger J, Cohen IL (2013). Prevalence of psychotropic drug use in adults with intellectual disability: positive and negative findings from a large scale study. J Autism Dev Disord.

[22] Eady N, Courtenay K, Strydom A (2015). Pharmacological management of behavioral and psychiatric symptoms in older adults with intellectual disability. Drugs Aging.

[23] SFS1993:387: Act Concerning Support and Service for Persons with Certain Functional Impairments (In Swedish: Lag om stöd och service till vissa funktionshindrade (LSS)). In*.* Stockholm, Sweden; 1993.

[24] WHO. Guidelines for ATC classification and DDD assignment. 2013.

[25] Chitty KM, Evans E, Torr JJ, Iacono T, Brodaty H, Sachdev P, Trollor JN (2016). Central nervous system medication use in older adults with intellectual disability: results from the successful ageing in intellectual disability study. Aust N Z J Psychiatry.

[26] Sheehan R, Hassiotis A, Walters K, Osborn D, Strydom A, Horsfall L (2015). Mental illness, challenging behaviour, and psychotropic drug prescribing in people with intellectual disability: UK population based cohort study. BMJ.

[27] McGuire BE, Daly P, Smyth F (2010). Chronic pain in people with an intellectual disability: under-recognised and under-treated?. J Intellect Disabil Res.

[28] Evenhuis H, Henderson CM, Beange H, Lennox N, Chicoine B (2001). Healthy ageing - adults with intellectual disabilities: physical health issues. J Appl Res Intellect Disabil.

[29] McCarthy J, O'Hara J (2011). Ill-health and intellectual disabilities. Curr Opin Psychiatry.

[30] Di Giorgio C, Provenzani A, Polidori P (2016). Potentially inappropriate drug prescribing in elderly hospitalized patients: an analysis and comparison of explicit criteria. Int J Clin Pharm.

[31] Voigt K, Gottschall M, Koberlein-Neu J, Schubel J, Quint N, Bergmann A (2016). Why do family doctors prescribe potentially inappropriate medication to elderly patients?. BMC Fam Pract.

[32] Axmon A, Björne P, Nylander L, Ahlström G. Psychiatric diagnoses in older people with intellectual disability in comparison with the general population: a register study. Epidemiol Psychiatr Sci 2017;Feb. 23:1–13. 10.1017/S2045796017000051 PMC6137377 28228177

[33] Maust DT, Kales HC, Wiechers IR, Blow FC, Olfson M. No End in Sight: Benzodiazepine Use in Older Adults in the United States. J Am Geriatr Soc. 2016:n/a-n/a. 10.1111/jgs.14379 PMC5173408 27879984

[34] Dehlin O, Bengtsson C, Rubin BA (1997). Comparison of zopiclone and propiomazine as hypnotics in outpatients: a multicentre, double-blind, randomized, parallel-group comparison of zopiclone and propiomazine in insomniacs. Curr Med Res Opin.

[35] Beuscart JB, Genin M, Dupont C, Verloop D, Duhamel A, Defebvre MM, Puisieux F (2017). Potentially inappropriate medication prescribing is associated with socioeconomic factors: a spatial analysis in the French Nord-pas-de-Calais region. Age Ageing.

[36] Napolitano F, Izzo MT, Di Giuseppe G, Angelillo IF (2013). Frequency of inappropriate medication prescription in hospitalized elderly patients in Italy. PLoS One.

[37] Emerson E (2012). Deprivation, ethnicity and the prevalence of intellectual and developmental disabilities. J Epidemiol Community Health.

[38] Koritsas S, Iacono T (2016). Weight, nutrition, food choice, and physical activity in adults with intellectual disability. J Intellect Disabil Res.

[39] Projovic I, Vukadinovic D, Milovanovic O, Jurisevic M, Pavlovic R, Jacovic S, Jankovic S, Stefanovic S (2016). Risk factors for potentially inappropriate prescribing to older patients in primary care. Eur J Clin Pharmacol.

[40] Zaal RJ, van der Kaaij AD, Evenhuis HM, van den Bemt PM (2013). Prescription errors in older individuals with an intellectual disability: prevalence and risk factors in the healthy ageing and intellectual disability study. Res Dev Disabil.

[41] Chutka DS, Takahashi PY, Hoel RW (2004). Inappropriate medications for elderly patients. Mayo Clin Proc.

[42] Keith SW, Maio V, Dudash K, Templin M, Del Canale SA (2013). Physician-focused intervention to reduce potentially inappropriate medication prescribing in older people: a 3-year, Italian, prospective, proof-of-concept study. Drugs Aging.

[43] Mattison ML, Afonso KA, Ngo LH, Mukamal KJ (2010). Preventing potentially inappropriate medication use in hospitalized older patients with a computerized provider order entry warning system. Arch Intern Med.

[44] Zaal RJ, Ebbers S, Borms M, Koning B, Mombarg E, Ooms P, Vollaard H, van den Bemt PM, Evenhuis HM (2016). Medication review using a systematic tool to reduce inappropriate prescribing (STRIP) in adults with an intellectual disability: a pilot study. Res Dev Disabil.

[45] Scheifes A, Egberts TC, Stolker JJ, Nijman HL, Heerdink ER (2016). Structured medication review to improve pharmacotherapy in people with intellectual disability and Behavioural problems. Journal of applied research in intellectual disabilities : JARID..

[46] Havercamp SM, Scandlin D, Roth M (2004). Health disparities among adults with developmental disabilities, adults with other disabilities, and adults not reporting disability in North Carolina. Public Health Rep.

[47] McGuire BE, Defrin R (2015). Pain perception in people with down syndrome: a synthesis of clinical and experimental research. Front Behav Neurosci.

[48] Walsh M, Morrison TG, McGuire BE (2011). Chronic pain in adults with an intellectual disability: prevalence, impact, and health service use based on caregiver report. Pain.

[49] Amor-Salamanca A, Menchon JM. Pain underreporting associated with profound intellectual disability in emergency departments. J Intellect Disabil Res. 2017; 10.1111/jir.12355 28054733

[50] Alzner R, Bauer U, Pitzer S, Schreier MM, Osterbrink J, Iglseder B (2016). Polypharmacy, potentially inappropriate medication and cognitive status in Austrian nursing home residents: results from the OSiA study. Wien Med Wochenschr.

[51] Fain KM, Alexander GC, Dore DD, Segal JB, Zullo AR, Castillo-Salgado C. Frequency and Predictors of Analgesic Prescribing in U.S. Nursing Home Residents with Persistent Pain. J Am Geriatr Soc. 2016:n/a-n/a. 10.1111/jgs.14512 PMC9588418 28198563

[52] Herr M, Grondin H, Sanchez S, Armaingaud D, Blochet C, Vial A, Denormandie P, Ankri J. Polypharmacy and potentially inappropriate medications: a cross-sectional analysis among 451 nursing homes in France. Eur J Clin Pharmacol. 2017; 10.1007/s00228-016-2193-z 28093640

[53] Qian J, Wittayanukorn S, McGuffey G, Hansen R (2015). Factors associated with psychotropic prescriptions, psychiatric hospitalization, and spending among Medicare beneficiaries under 65. Disabil Health J.

[54] Mo L, Ding D, SY P, Liu QH, Li H, Dong BR, Yang XY, He JH (2016). Patients aged 80 years or older are encountered more potentially inappropriate medication use. Chin Med J.

[55] Murphy Y, Wilson E, Goldner EM, Fischer B (2016). Benzodiazepine use, misuse, and harm at the population level in Canada: a comprehensive narrative review of data and developments since 1995. Clin Drug Investig.

[56] WH L, Wen YW, Chen LK, Hsiao FY (2015). Effect of polypharmacy, potentially inappropriate medications and anticholinergic burden on clinical outcomes: a retrospective cohort study. CMAJ.

[57] Henschel F, Redaelli M, Siegel M, Stock S (2015). Correlation of incident potentially inappropriate medication prescriptions and hospitalization: an analysis based on the PRISCUS list. Drugs Real World Outcomes.

[58] Ni Chroinin D, Neto HM, Xiao D, Sandhu A, Brazel C, Farnham N, Perram J, Roach TS, Sutherland E, Day R (2016). Potentially inappropriate medications (PIMs) in older hospital in-patients: prevalence, contribution to hospital admission and documentation of rationale for continuation. Australas J Ageing.

[59] Endres HG, Kaufmann-Kolle P, Steeb V, Bauer E, Bottner C, Thurmann P (2016). Association between potentially inappropriate medication (PIM) use and risk of hospitalization in older adults: an observational study based on routine data comparing PIM use with use of PIM alternatives. PLoS One.

[60] Meurer WJ, Potti TA, Kerber KA, Sasson C, Macy ML, West BT, Losman ED (2010). Potentially inappropriate medication utilization in the emergency department visits by older adults: analysis from a nationally representative sample. Acad Emerg Med.

[61] Fu AZ, Jiang JZ, Reeves JH, Fincham JE, Liu GG, Perri M, 3rd. Potentially inappropriate medication use and healthcare expenditures in the US community-dwelling elderly. Med Care 2007;45(5):472–476. 10.1097/01.mlr.0000254571.05722.34 17446834

[62] Counter D, Stewart D, MacLeod J, McLay JS. Multicompartment compliance aids in the community: the prevalence of potentially inappropriate medications. Br J Clin Pharmacol. 2017;83(7):1515-20. doi:10.1111/bcp.13220. Epub 2017 Jan 31. 10.1111/bcp.13220 PMC5465329 28009450

[63] Kachru N, Carnahan RM, Johnson ML, Aparasu RR (2015). Potentially inappropriate anticholinergic medication use in community-dwelling older adults: a national cross-sectional study. Drugs Aging.

[64] Morgan SG, Weymann D, Pratt B, Smolina K, Gladstone EJ, Raymond C, Mintzes B (2016). Sex differences in the risk of receiving potentially inappropriate prescriptions among older adults. Age Ageing.

